# The Role of the Medial Septum—Associated Networks in Controlling Locomotion and Motivation to Move

**DOI:** 10.3389/fncir.2021.699798

**Published:** 2021-07-22

**Authors:** Petra Mocellin, Sanja Mikulovic

**Affiliations:** ^1^Department of Cellular Neuroscience, Leibniz Institute for Neurobiology, Magdeburg, Germany; ^2^International Max Planck Research School for Brain and Behavior, Bonn, Germany; ^3^Research Group Cognition and Emotion, Leibniz Institute for Neurobiology, Magdeburg, Germany

**Keywords:** MSDB, locomotion, motivation, theta, cell types

## Abstract

The Medial Septum and diagonal Band of Broca (MSDB) was initially studied for its role in locomotion. However, the last several decades were focussed on its intriguing function in theta rhythm generation. Early studies relied on electrical stimulation, lesions and pharmacological manipulation, and reported an inconclusive picture regarding the role of the MSDB circuits. Recent studies using more specific methodologies have started to elucidate the differential role of the MSDB’s specific cell populations in controlling both theta rhythm and behaviour. In particular, a novel theory is emerging showing that different MSDB’s cell populations project to different brain regions and control distinct aspects of behaviour. While the majority of these behaviours involve movement, increasing evidence suggests that MSDB-related networks govern the motivational aspect of actions, rather than locomotion *per se*. Here, we review the literature that links MSDB, theta activity, and locomotion and propose open questions, future directions, and methods that could be employed to elucidate the diverse roles of the MSDB-associated networks.

## Introduction

Movement, and above all locomotion, is essential for most species’ survival: we move to reach a specific place, receive a reward, flee from a predator or attack prey. However, while extensive research has been conducted on brain circuits underlying locomotion (Sinnamon et al., 1987; Fuhrmann et al., 2015; Howe and Dombeck, 2016; Capelli et al., 2017; Justus et al., 2017; Caggiano et al., 2018), surprisingly less attention has been paid to the influence that the internal state of a subject may have on specific motor performances (Mogenson et al., 1980; Ferreira-Pinto et al., 2018). For example, the intensity at which a movement is performed may arise from a cumulative integration of individual sensory modalities (Bland and Oddie, 2001) or it may be based on experience and thus be retrieved from memory. Likewise, the decision to act or not is shaped by both the external and the internal environment, a combination of external inputs, intrinsic drive, and visceral homeostasis. This implies that similar motor outputs could result from different motivations and intentions guided by different brain regions and/or circuits.

**FIGURE 1:**
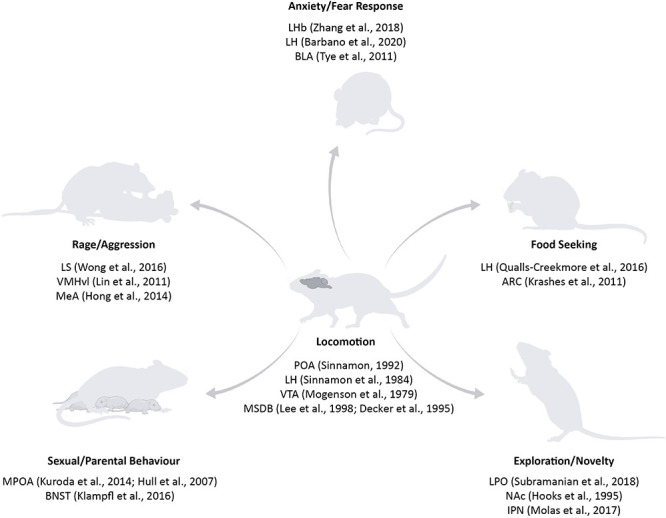
Subcortical structures involved in locomotion and in different motivations to move. Key areas in the basal forebrain, hypothalamic, and midbrain structures have been classically linked to locomotion (for example, POA, LH, VTA, and MSDB) (Mogenson et al., 1979; Sinnamon et al., 1984; Lee et al., 1988; Sinnamon, 1992; Decker et al., 1995). With the rise of new tools, it was possible to specify the contribution of specific areas on distinct reasons to move: fear responses are controlled by BLA, LHb, and LH (Tye et al., 2011; Zhang et al., 2018; Barbano et al., 2020); food seeking centres have been found in the LH and ARC (Krashes et al., 2011; Qualls-Creekmore and Münzberg, 2018); exploration and novelty are mediated by LPO, NAc, and IPN (Hooks and Kalivas, 1995; Molas et al., 2017; Subramanian et al., 2018); sexual and parental behaviour are mostly related to POA and BNST (Hull and Dominguez, 2007; Kuroda and Numan, 2014; Klampfl et al., 2016); rage and aggression nuclei include LS, VMHvl, and MeA (Lin et al., 2011; Hong et al., 2014; Wong et al., 2016). POA, Preoptic Area; LH, Lateral Hypothalamus; VTA, Ventral Tegmental Area; MSDB, Medial Septum and Diagonal Band of Broca; BLA, Basolateral Amygdala; LHb, Lateral Habenula; ARC, Arcuate Nucleus; LPO, Lateral Preoptic Area; NAc, Nucleus Accumbens; IPN, Interpeduncolar Nucleus; BNST, Bed Nucleus of the Stria Terminalis; LS, Lateral Septum; VMHvl, Ventromedial Nucleus of the Hypothalamus; MeA, Medial Amygdala. Source icons were used from @biorender.com.

The brain networks underlying the execution and planning of locomotion are widely spread throughout the central nervous system: spinal cord, hindbrain, midbrain, basal ganglia, and cortex (Garcia-Rill, 1986; Jordan et al., 2008; Okada and Okaichi, 2010; Kiehn, 2016). Certain structures are proven to be essential for locomotion, for example the central pattern generator in the spinal cord (Sherrington, 1910; Brown, 1914) or the mesencephalic locomotor region in the brainstem (Douglas et al., 1993), as their lesions lead to severe impairment in movement or even immobility. Other regions are actively involved prior and during locomotion: the motor cortex performs motor planning (Li et al., 2015), the striatum facilitates voluntary movement execution (Tang et al., 2007; Cui et al., 2013), and the cerebellum adjusts the action based on the environmental changes (Robinson, 1995). Despite the significant amount of work already conducted to understand the neural basis of movement and how the signal is transmitted from the neurons in the central nervous system to the muscles, we still do not understand “where” the decision to start moving is formed in the brain. Seminal studies performed in the 70s demonstrated that electrical stimulation of a great variety of brain regions can ultimately lead to movement: the Medial Septum and diagonal Band of Broca (MSDB), the basal forebrain bundle, the hypothalamic nuclei and the ventral tegmental area (VTA) are among the most studied circuits (Mogenson et al., 1979, 1980; Parker and Sinnamon, 1983; Sinnamon et al., 1984, 1987; Lee et al., 1988; Decker et al., 1995). However, as previously mentioned, an animal can move for different reasons and we know that the activity of different areas drives specific motivation to move. The preoptic area is involved in movement linked to parental, sexual, and maternal behaviour (Noonan and Kristal, 1979; Gorski, 1984; Hull and Dominguez, 2007; Kuroda and Numan, 2014), the lateral septum (LS) mediates rage and attack towards conspecifics (Wong et al., 2016), the hypothalamus plays a role in food seeking behaviour (Qualls-Creekmore et al., 2017) and the MSDB is mostly linked to navigation (Brandon et al., 2011; Koenig et al., 2011; Wang et al., 2015), but also other movement-related behaviours including exploration, anxiogenic and anxiolytic locomotion (Figure 1). While the advances in techniques and methods to manipulate and study neurons have tremendously developed in the last two decades, the unsupervised sub-second analysis of the behavioural readout has just recently started to attract the neuroscience field’s attention (Hausmann et al., 2021).

The MSDB is one of the most interconnected regions of the brain given its key position in the middle of the basal forebrain. Among others, it receives inputs from thalamus, supramammillary nuclei (SUM), VTA, *nucleus incertus* (NI), and cerebellum (Ang et al., 2017; Müller and Remy, 2018; Watson et al., 2019), and projects back to both dorsal and ventral hippocampus (HPC), cingulate and insular cortex, hypothalamus, habenula, and VTA (Swanson and Cowan, 1979; Fuhrmann et al., 2015; Ang et al., 2017). The MSDB has been considered a central subcortical hub for information processing, and it has been intensively studied for its high number of cholinergic neurons. In fact, for several years, MSDB was thought to comprise only two different kinds of cells: the cholinergic ones, positive for the choline acetyltransferase (ChAT) enzyme, and the inhibitory interneurons. Only at the beginning of the 21st century, a third subpopulation was described: the glutamatergic neurons that express transcripts for the vesicular glutamate transporter 2 (VGluT2; Sotty et al., 2003) and do not overlap with the cholinergic nor the GABAergic populations. MSDB has been implicated in numerous behaviours related to movement including cognitive tasks (Colom et al., 2005; Mamad et al., 2015; Wang et al., 2015; Jacob et al., 2017), locomotor (Bland and Oddie, 2001; Bland et al., 2007; Fuhrmann et al., 2015; Justus et al., 2017; Jin et al., 2019), and emotional responses (Highfield et al., 2000; Khakpai et al., 2013; Knox and Keller, 2016; Jiang et al., 2018). It has also been historically related to theta rhythm (4–12 Hz), a distinctive oscillatory activity that can be either recorded in the HPC of anesthetised and immobile animals (so called type 2 theta), or during locomotor behaviour (so called type 1 theta) (Kramis et al., 1975). Interestingly, lesions or pharmacological silencing of MSDB abolish theta oscillations in the HPC (Lee et al., 1994). A large body of studies in awake animals proved a link between theta activity and locomotion and revealed that MSDB silencing not only impacts the hippocampal theta rhythm, but also reduces the overall locomotor activity (Lee et al., 1988; Decker et al., 1995; Fraser et al., 1991). However, the development of more sophisticated approaches like chemo- and optogenetic manipulations reported little effect on locomotion following MSDB inhibition (Sweeney et al., 2017). These conflicting results can be partially explained by the indistinct and generalised silencing of a brain area in pharmacological or lesion studies, versus the more accurate and precise inhibition achieved with modern techniques. Indeed, optogenetic experiments carried in rodent Cre lines and targeting specific MSDB’s subpopulations (Fuhrmann et al., 2015; Zhang et al., 2018) have shown that the observed locomotor output depends on the activation of the septal glutamatergic neurons. We here review the literature that links MSDB, theta activity, and locomotion, with a focus on the overlooked fact that an animal moves for different reasons and with different motivations. We list open questions and future directions, as well as methods that could be employed to elucidate the diverse behavioural roles of the MSDB-related networks.

## Early Behavioural Studies With Septal Electrical Stimulation or Electrolytic Lesions

For decades, electrical stimulation or lesions of the septal area were the only ways experimenters possessed to understand the physiological role of this basal forebrain region. Electrical stimulation of the septal area showed, from the very beginning, a broad range of effects: in awake animals it produced positive reinforcement (Olds and Milner, 1954), no reduction of food intake (Mabry and Peeler, 1968), but reduction of saline preference (Gentil et al., 1971) and of water intake (Wishart and Mogenson, 1970). An increase in shaking, grooming and feeding was observed after stimulation (Altman and Wishart, 1971), as well as an increase in self-stimulation (Gordon and Johnson, 1981; Cazala et al., 1988), hyperactivity, and yawning (Wishart et al., 1973). However, when applying electrical stimulation, it is cumbersome to define the volume of the affected area and it remains unknown which brain regions are sending the input or receiving the electrical output. Furthermore, it remains unclear whether the targeted area is necessary or just involved in the observed behavioural process (Vaidya et al., 2019). For these reasons, using lesions that permanently destroy the area of interest may offer more relevant information in relation to the studied behaviour than the electrical activation of the circuit.

The first seminal work describing the behavioural effects following septal lesions dates back to 1953 (Brady and Nauta, 1953). Building on few previous studies performed in cats (Spiegel et al., 1940), this rodent based study hypothesised a role for the septum in emotional and affective behaviour. Animals with septal lesions showed increased rage and startle responses when handled by the experimenter or presented with auditory stimuli (Caplan, 1973). Even innocuous stimuli leading to exploration in non-lesioned animals were eliciting freezing or attacks in the lesioned ones. However, while apparently any stimulus would lead to exaggerated responses, it was reported that the same animals were less anxious and fearful than the controls when placed back in the chambers where they were conditioned with a foot shock prior to the surgery (Caplan, 1973). These seemingly contradictory results showing both hyperreactivity and anxiolytic effects after septal lesions promoted a series of other studies trying to elucidate the role of the septal area and the contribution of its subregions to these behavioural paradigms. In the meantime, an increasing amount of data accumulated and raised the idea of the so called “septal syndrome,” septal hyperreactivity or septal hyperemotionality not only in mice and rats, but also in hamsters, cats, and monkeys (Brady and Nauta, 1955; King, 1958; Votaw, 1960; Lubar, 1964; McCleary et al., 1965; Buddington et al., 1967; Sodetz et al., 1967). The core results appointed the septal circuits as inhibitory networks suppressing responses to stimuli with a predominant role in negatively reinforced behaviours. Thus, septal lesions disinhibit and enhance the animal responsivity to negative events, while also leading to a lack of response suppression in avoidance tasks (Caplan, 1973). It is, however, important to note that the results obtained in the abovementioned experiments resulted from a general lesion of the basal forebrain with no specificity for the MSDB or LS, and included also the bed nucleus of the *stria terminalis*. When lesions were targeted to the MSDB only, it became evident that the major role in the septal syndrome was played by the LS, with a minor contribution of the MSDB in enhancing the aggressive behaviour and the animal’s emotionality (Poplawsky and Johnson, 1973; Albert and Richmond, 1975; Albert and Chew, 1980; Lee et al., 1988). These data were confirmed in a more recent study showing that LS GABAergic projections to the ventromedial hypothalamus are necessary to suppress male aggression in mice. When the same projections are inhibited, the number of attacks to both males and females increased and a “septal rage” behaviour was reported (Wong et al., 2016).

A different picture arose when focussing on lesions of the MSDB specifically: MSDB disruption resulted in submissive behaviour with conspecifics (Poplawsky and Johnson, 1973), decreased horizontal and vertical movements when measuring exploratory and locomotor behaviour (Lee et al., 1988) slower rate of habituation (Decker et al., 1992), deficits in spatial tasks (Fraser et al., 1991), decreased spatial discrimination and increased time in the open arm of the elevated plus maze (Decker et al., 1995). If some of these effects (deficits in exploration, locomotion, and habituation) were expected in light of the strong projections of the MSDB to the HPC formation and their involvement in navigation and memory (Pang et al., 2011; Mamad et al., 2015; Hinman et al., 2018); other results like the submissive behaviour and the anxiolytic effect are less easy to interpret. Moreover, several studies report impaired exploratory and locomotor skills of MSDB-lesioned animals only in the first post-operative trial, showing a slower but constant increase in performances over days until the point of being not significantly different from controls (for example in Myhrer, 1989). Possible explanations behind these phenomena can be identified in increased anxiety of MSDB-lesioned animals when exposed to novel environments (neophobia), deficits in sensory information processing and/or in short- and long-term memory (thus inability to recognise a novel environment and explore it), fastest stimulus satiation or increased inertia. Overall, early investigations of the MSDB highlighted a very close relationship between this brain area and locomotion. Nevertheless, it remained unclear what was the exact contribution of the MSDB in driving and/or modulating movement-related responses. Relevant to the interpretation of these findings is the fact that all reported studies were performed in rats and not in mice. Compared to mice, rats are predator and not prays, they show more prominent social interactions and more advanced cognitive processing allowing them to solve complex tasks (Scott, 1966). They also differ in terms of genetic background, gene expression, and ion channels, making it cumbersome to compare the behavioural effects of MSDB manipulation between the two species (Bonthuis et al., 2010; Hok et al., 2016). In addition, these studies used electrolytic lesions of the septal area, meaning that not only the somata, but also the axonal fibres crossing the region, were affected. Moreover, permanent lesions of a brain area may lead to reorganisation of the system and changes in the homeostatic activity of the network, thus the timing (immediate vs weeks or months after the lesions) at which the studies were conducted are relevant to properly interpret the lesions’ effect on the underlying behavioural output.

## Pharmacological Manipulation of the MSDB

From the beginning of the 80s pharmacology emerged as a new tool to silence specific brain areas. Agonists and antagonists of ion channels and receptors became a widely used means to investigate the effects of blocking specific neurotransmitters and allowed the silencing of areas with only small effects on the *en passant* fibre tracts. Indeed, muscimol infusion only blocks cell bodies, while local anaesthetics and tetrodotoxin can also block the passing fibres (Martin and Ghez, 1999). The latter can affect projecting axons originating from cortical and forebrain structures. Those projections take part in the information flow from the MSDB through three different pathways: to the hippocampus via the dorsal fornix fimbria; to the habenular nuclei through the stria medullaris; and via the medial forebrain bundle running ventral into the thalamic and hypothalamic regions, crossing the midbrain and reaching the brainstem (Meibach and Siegel, 1977).

Muscimol (GABA A agonist) and lidocaine (Na^+^ channels blocker) were used to temporarily silence or reduce the activity in the targeted brain region through two opposite mechanisms: increasing inhibitory interneuron activity through disinhibition and reducing action potential firing probability, respectively. When applied in the MSDB, the most striking effect on the animal’s behaviour was the impairment in tasks requiring memory and navigation (Chrobak et al., 1989; Nagahara and McGaugh, 1992; Walsh et al., 1998), confirming the crucial importance of septal projections to the HPC formation. MSDB inhibition via lidocaine seemed not to affect the running speed (Koenig et al., 2011), but to reduce anxiety and increase open arm exploration in a plus maze (Lamprea et al., 2010). Muscimol infusions resulted in more diversified and sometimes contradictory behaviours. Upon muscimol infusion, mice displayed both increased arousal and locomotor activity (Osborne, 1994), or slightly decreased running speed (Wang et al., 2015) or even no effect on locomotion (Brandon et al., 2011) depending on the experimental conditions. Indeed, muscimol leads to the specific excitation of GABAergic neurons in the MSDB; this, in turn, could lead to the activation of different pathways related to either arousal (Wu et al., 2002), nociception (Ang et al., 2015), anxiety (Vickstrom et al., 2020), or reward-seeking behaviour, as demonstrated by an increase of lever presses for muscimol self-administration (Gavello-Baudy et al., 2008). Thus, it is not surprising that the use of this drug leads to a diverse repertoire of behavioural outcomes.

Alongside muscimol and lidocaine, a wide variety of other manipulation approaches have also been studied in relation to MSDB and locomotion. For example, infusion of histamine or pyrilamine increased locomotion (Zarrindast et al., 2006), depletion of the relaxin-family peptide-3 receptor (RXFP3) impaired spatial strategy search (Haidar et al., 2019), the neurokinin 1 receptor (NK1R) facilitated exploratory behaviour (Ng et al., 2020), while CaV3.1 (T-type voltage gated calcium channel) knock down increased exploration of an object (Jung et al., 2019). Moreover, MK 801 and ketamine lead to hyperlocomotion (Ma and Leung, 2007), while a somatostatin-sensitive mechanism facilitated inactivity periods in open field (Ng et al., 2020). A recent study applied the cooling of MSDB as an alternative and complementary tool to pharmacology in order to investigate the overall effect of the MSDB circuit inhibition. The authors showed a reduction of theta rhythm upon cooling as well as an increase in number of choice errors in a spatial navigation task (Petersen and Buzsáki, 2020). While these results are valuable to investigate the general aspects of MSDB circuit function and neuromodulation, the specific contribution of the distinct cell types within the MSDB was not investigated until the development of Cre-dependent manipulation in transgenic mouse lines.

## Cell Type Specific Manipulations of the MSDB

The first studies using Cre lines focussed on the impact of cholinergic (ACh) and GABAergic septal neurons on animal behaviour.

Choline acetyltransferase (ChAT)-Cre transgenic mice have been considered the gold standard for ACh neurons targeting. Chemogenetic silencing of these neurons in the MSDB alleviates pain induced anxiety (Jiang et al., 2018) and produces a general anxiolytic effect corroborated by an increase in distance travelled in the open field test and higher open arms entrances in the elevated plus maze test (Zhang et al., 2017). Thus, ACh MSDB transmission seems to promote anxiety possibly through its projections to the ventral HPC and the prefrontal cortex (Adhikari et al., 2010; Mikulovic et al., 2018). This finding could partially reconcile the reduced locomotor activity and anxiety-like behaviour described by some early lesions studies (Caplan, 1973), while reproducing the increased time in the open arm and exploratory behaviour shown in others (Decker et al., 1992). We could speculate that non-specific lesions could differentially affect ACh transmission and projections outside the MSDB, thus eliciting opposite kinds of behaviours. Chemogenetic activation of ChAT positive cells in MSDB reduces frequency of theta oscillations in the entorhinal cortex and gives rise to the sense of novelty and anxiety by increasing the time of immobility and avoidance of the centre (Carpenter et al., 2017). In line with these observations, several studies (Adhikari et al., 2010, 2011; Mikulovic et al., 2018) have shown that anxiety-related behaviour induces slower theta rhythm, resembling the cholinergic-dependent type 2 theta, particularly in the ventral HPC. Interestingly, depending on the context, type 2 theta may underlie anxiogenic or anxiolytic behaviour. For example, in open field or elevated plus maze anxiety-related tests (Adhikari et al., 2010, 2011) cholinergic-dependent type 2 theta relates to the increased anxiety and reduction in locomotion. Differentially, in a predator odour test (Mikulovic et al., 2018), type 2 theta underlies increased risk-taking behaviour and locomotion. These results indicate that the behavioural effect of the type 2 theta might depend on the arousal level, commonly related to the acetylcholine levels (Pepeu and Giovannini, 2004).

GABAergic neurons comprise a wide variety of cell types expressing different proteins such as parvalbumin (PV), somatostatin (SST), or calretinin. Specific modulation of GABAergic neurons through Gad65-Cre, Gad67-Cre, PV-Cre, or SST-Cre lines elucidated the role of interneurons in the MSDB network showing how different projection targets give rise to different behaviours. GABAergic MSDB neurons densely project to the HPC formation and are strictly linked to theta generation not only during movement, but also during rest and sleep. Manipulation of the GABAergic septal-hippocampal network has an impact on memory discrimination (Salib et al., 2019) and sequential learning (Dwyer et al., 2007) most probably due to the disruption of internally generated theta oscillations. Activation of these neurons during rapid-eye-movement (REM) phases also affects spatial and contextual memory consolidation in mice (Boyce et al., 2016). On the other hand, activation of MSDB interneurons was reported to increase object exploration in awake animals and type 2 theta rhythm in anaesthetised animals, without affecting open field exploration (Gangadharan et al., 2016). PV^+^ cells in the MSDB overlap with the population of the hyperpolarization-activated cyclic nucleotide-gated (HCN) channel expressing neurons and present pacemaker activity responsible for theta entrainment in the MSDB (Varga et al., 2008). SST neurons instead comprise a small population of basal forebrain neurons, which in the MSDB appear to be responsible for spatial working memory. When photo-inhibited, they do not affect the animal speed but instead disrupt the alternation index in a Y maze test (Espinosa et al., 2019). Finally, little is known about MSDB interneurons’ input and output connectivity outside the MSDB and HPC formation, but it has been shown that GABAergic transmission to the MHb is sufficient to entrain the local circuit firing (Choi et al., 2016) and seems to mediate an anxiogenic and depressive state of the animal modulated by the endocannabinoid signalling (Vickstrom et al., 2020).

Glutamatergic (VGluT2^+^) neurons were only recently described as part of the MSDB circuit. They are mostly located in the septum midline and in the diagonal band of Broca, and they are highly interconnected (Manseau et al., 2005). While the majority of the early studies (Manns et al., 2001; Hajszan et al., 2004; Colom et al., 2005) had focussed on their electrophysiological and molecular characteristics, several recent studies tried to elucidate the involvement of this cell type in behaviour. Optogenetic stimulation of these neurons leads to entraining of theta oscillations in the HPC (Robinson et al., 2016), locomotor activity (Fuhrmann et al., 2015), and appetite suppression (Sweeney et al., 2017). In particular, this is the only cell type in MSDB whose activation leads to nearly instantaneous locomotion that lasts for several seconds following the stimulus offset (Fuhrmann et al., 2015). It has been recently shown (Korvasová et al., 2021) that the locomotor effect that ensues upon MSDB VGluT2^+^ cell stimulation does not require theta oscillations, nor relies upon local MSDB connectivity, given that locomotion effect persists even when the synaptic connectivity in MSDB is completely blocked. Furthermore, the persistent locomotion is linked to intrinsically generated persistent firing of the MSDB VGluT2^+^ neurons.

Tracing studies (Fuhrmann et al., 2015; Agostinelli et al., 2017; Zhang et al., 2018) have shown that MSDB VGluT2^+^ neurons project to different brain areas: the HPC, the LHb, POA, the paraventricular (PVH), lateral (LH), and posterior (PH) hypothalamic nuclei, the SUM, the VTA, the NI, and the raphe nucleus. Interestingly, manipulation of glutamatergic projections in each of these target areas exerted a different effect. In the LHb it caused place aversion without affecting the locomotor activity (Zhang et al., 2018) that seemed to be mediated by POA projections (Zhang et al., 2018). MSDB inputs to LH have been involved in arousal: their optogenetic activation promotes wakefulness and theta power, while their silencing increased NREM sleep (Manseau et al., 2005). This network seems to be also related to reinforcement and motivation through MSDB inputs to the VTA. When self-stimulating these projections, the animals will increase lever pressing and this action in turn increases nucleus accumbens (NAc) DA release (Kesner et al., 2020), classically associated to rewarding mechanisms.

Overall, it seems clear that MSDB cell types and their faceted projections exert a quite broad effect on animal behaviour. ChAT neurons are strongly projecting to the vHPC and are involved in anxiogenic and anxiolytic responses. They may play a role in guiding an animal’s action based on its internal state, thus modifying locomotor responses based on possible threatening stimuli present in the environment (Mikulovic et al., 2018). GABAergic septal interneurons, on the other side, are highly interconnected inside the MSDB and with the HPC formation (Freund and Antal, 1988; Gonzalez-Sulser et al., 2014; Salib et al., 2019; Schlesiger et al., 2021), for this reason they play a major role in pace making activity and theta generation. To our knowledge, they do not send dense projections to other cortical or subcortical regions apart from the HPC formation and some related structures like the retrosplenial cortex (Unal et al., 2015) and their role in behaviour has not been deeply investigated so far. These interneurons appear to be active in aversive conditions as for example nociception, anxiety and depressive states, or in promoting arousal, that can be linked to an increase in alert and awareness for the animal to be ready to react. Finally, VGluT2^+^ neurons are the group of MSDB cells more strongly related to movement. Their optogenetic activation induces locomotion and reinforcement effects. Depending on their output region they may mediate place aversion (as for example when activating the LHb), purely locomotion (as for POA projections) or wakefulness (through their inputs to LH). Their role in behaviour is still under investigation but the data collected so far allow speculating for a key involvement of these neurons in action initiation. The fact that their stimulation on the somata or on the projections increase the overall arousal of the animals and leads them to move, shows the VGluT2^+^ MSDB neurons as possible candidates to mediate “fast” responses to the context the animal is in, as opposed to the LS mediated responses that appear to require more time as they must integrate more diversified inputs (Wirtshafter and Wilson, 2021).

How these three cell populations interact, and how their cross-talk can influence an animals’ behaviour is still an open question. However, several studies have focussed on their interplay associated with the generation and modulation of theta rhythm, locally and in target structures of the MSDB.

## MSDB, Theta Activity, and Locomotion Planning

While initially studied in relation to locomotion, MSDB has been in the last five decades mostly investigated in relation to theta oscillations in the HPC, as MSDB lesions or pharmacological inactivation abolished theta rhythm (Petsche and Stumpf, 1960; Petsche et al., 1962; Donovick, 1968; Gray, 1971; Kramis et al., 1975; Andersen et al., 1979; Buzsáki et al., 1986; Kocsis et al., 1999; Brandon et al., 2011; Koenig et al., 2011; Müller and Remy, 2018; Zutshi et al., 2018). However, a long-standing question is how these functions are associated and act together in the generation of behavior. Early studies (Yoshii et al., 1966; Vanderwolf, 1968) have shown that theta oscillations can be recorded in the HPC during voluntary motor behaviour such as walking, running, jumping, rearing, swimming, and digging, the so called type 1 behaviours (Pickenhain and Klingberg, 1967; Vanderwolf, 1968; Whishaw and Schallert, 1977). In contrast, during motor behaviours such as chewing, licking, grooming, and shivering, theta rhythm is absent and a large-amplitude irregular field activity (LIA) is recorded in the HPC. These behaviours are called type 2 or automatic behaviours (Vanderwolf, 1968; Sainsbury, 1970). Theta frequency was reported to increase as a function of speed (Bland and Vanderwolf, 1972; Hinman et al., 2011, 2016; Gupta et al., 2012; Winter et al., 2015), while more recent observation in rats reported mostly correlation with acceleration (Kropff et al., 2021). Theta amplitude correlates with the vigour (e.g., theta amplitude during a run or jump is higher than during a walk) (Whishaw and Vanderwolf, 1973). Significant body of evidence suggests that different types of theta rhythms in the HPC, driven by different MSDB inputs (cholinergic, GABAergic or glutamatergic), accompany different types of movement-related behaviours. The so-called type 1 theta, characterised by a higher oscillatory frequency (8–12 Hz), accompanies type 1 behaviours and it is controlled by the glutamatergic MSDB neurons (Fuhrmann et al., 2015). MSDB VGluT2^+^ neurons fire mostly tonically during theta oscillations and their optogenetic stimulation drives theta activity and locomotion speed in a frequency dependent manner (Fuhrmann et al., 2015). Differently, a small subset of MSDB GABAergic neurons expressing PV display highly rhythmical discharge, phase locked to the ongoing theta oscillations (Kocsis et al., 2021) and their optogenetic activation controls the oscillatory frequency outside the endogenous theta range and does not affect locomotion (Zutshi et al., 2018). Those neurons are thus commonly referred to be the pacemakers of theta activity (Zutshi et al., 2018; Kocsis et al., 2021). The less explored type of theta oscillations is type 2 theta, proven to be dependent on the cholinergic MSDB system. This rhythm has been mostly observed in immobile animals that are in a sensory processing mode (Kramis et al., 1975; Kramis and Routtenberg, 1977; Bland et al., 1984). Several stimuli were reported to induce type 2 theta, including olfactory, visual, auditory, and tactile stimulation. Type 2 theta appearance habituates following the repeated representation of the stimulus (Sainsbury and Montoya, 1984) and it has been proposed to code for the movement that follows. Brian Bland, one of the pioneers on the studies of type 2 theta activity, has put forward the theoretical framework that this rhythm plays a major role in “sensorimotor integration” (Bland et al., 1984; Bland, 1986). This hypothesis is centred around the idea that the circuitry underlying theta rhythmogenesis continuously provides the updated information about the changing environmental conditions to the voluntary motor system. In other words, animals are permanently exposed to a number of sensory stimuli in the environment of which some are relevant for the survival, while others can be ignored. This implies that neural systems that process the sensory information must recruit appropriate motor responses in order to make the appropriate final decision.

One study (Oddie et al., 1997) has tested this hypothesis in a very interesting way. In their paradigm, Oddie et al. (1997) investigated a pair of hungry rats fighting for a piece of food. The eating rat was called “victim,” while the rat attempting to steal the food was named “robber.” Robber’s action was regarded as the eliciting stimulus, while dodging—the lateral evasive movement by the victim rat, was the measured behavioural response. The authors hypothesised that the decision to dodge requires considerable sensory integration and planning—robber’s location and approach, the size of the food, and the eating time. Thus, if the type 2 theta underlies the selection of an upcoming motor response, this rhythm should occur prior to the dodge initiation and infusion of atropine, a cholinergic antagonist, should disturb the dodging behaviour. Motor abnormalities—a “collapsed” eating posture and the inability to hold the food between the forelimbs—was reported in the first 5–10 min after atropine application. Prior to the animals’ dodging, the frequency of the recorded theta rhythm increased, while the infusion of atropine in the victim’s MSDB completely abolished theta activity during the robber’s s attempts to steal the food and affected its success in protecting it. Interestingly, once the robber had stolen the food from the victim, the victim was capable to engage as a robber in an attempt to retrieve his food back. The authors conclude that, as the effect seems to be specific to dodging, it is rather not motivational. However, if the motor planning was affected in general, it is not intuitive to expect that the victims would take the role of a robber without displaying locomotion impairments. In our opinion, type 2 theta, and thus the cholinergic system of MSDB relate to locomotion planning only of specific valence, allowing the victim to act once the robber steals its food, but not to protect it from the theft. In this view, it seems that atropine reduce the victim’s capability to predict the robber’s action, but did not impair its ability to act after the robbery occurred. Thus, it appears that this system is not linked with locomotion in general but with the specific motivation of an animal to move.

**FIGURE 2:**
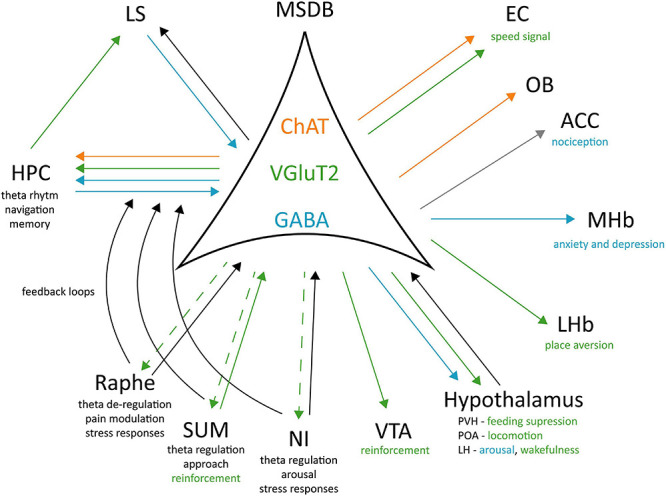
Cell-type specific connectivity of the MSDB. MSDB ChAT, VGluT2, and GABA neurons project to different brain regions and differently contribute to specific behaviours. Green arrows: VGluT2 projections; Blue arrows: GABAergic projections; Orange arrows: ChAT projections; Gray arrow: polysynaptic input; Black arrows: unspecified neurotransmitter; Dashed arrow: unknown physiological role of the projection. ChAT, Choline acetyltransferase; VGluT2, Vesicular Glutamate Transporter 2; GABA, Gamma aminobutyric acid; EC, Entorhinal Cortex; OB, Olfactory Bulb; ACC, Anterior Cingulate Cortex; MHb, Medial Habenula; LHb, Lateral Habenula; PVH, Posterior Ventral Hypothalamic nucleus; POA, Preoptic Area; LH, Lateral Hypothalamus; VTA, Ventral Tegmental Area; NI, Nucleus Incertus; SUM, Supramammillary Nucleus; LS, Lateral Septum; Hippocampus, HPC.

Furthermore, if type 2 theta codes the future movement planning, one would expect its presence also during locomotion, not solely during immobility. While Brian Bland has been postulating for years that type 1 and type 2 theta appear coincidentally (Bland and Oddie, 2001), the experimental evidence for it was lacking for a considerable amount of time. One recent study (Mikulovic et al., 2018) has shown that type 2 theta can originate in the ventral HPC. Indeed, ventral HPC receives strong cholinergic input from MSDB and is involved in emotional information processing, in contrast to its dorsal counterpart, known for its role in navigation and cognition (Strange et al., 2014). When animals are taking risks in an anxiety predator-odour test, type 2 theta co-exists with type 1-theta in the ventral HPC. These results additionally support our views that type 2 theta code for locomotion of specific valence. Several other studies support this hypothesis. In an early study (Whishaw and Vanderwolf, 1973), the authors have shown that in a jump avoidance test, where a rat learns to jump to avoid an electrical shock, the recorded theta activity, supposed to correspond to the type 2 theta rhythm, predicts the height of the jump. This study was replicated by Bland et al. (2006) while another study (Balleine and Curthoys, 1991) (discussed in Bland et al., 2007) added an interesting twist to this paradigm. They investigated three different conditions: escapable shock, non-escapable shock and no shock. The rats were trained to each of the condition on the first day and 24 h later tested, while the oscillatory activity was recorded prior to the shock. Interestingly, while escapable and no shock rats generated theta oscillations during immobility, LIA was recorded in the HPC of the inescapable rats. All this indicates that theta rhythm and the underlying MSDB circuit function as a readout for different movements and internal state of the subject. This information is subsequently transferred to the specific region that receives MSDB inputs, leading to a specific motivation to move (Figure 2). One brain region that is anatomically and functionally close and thus acts in synergy with MSDB is the LS.

## Synergy Between MSDB and LS

As discussed above, for a long time the septal area was studied as a whole, without paying attention to the different roles played by its medial and lateral part. Over the years, several evidences pointed to the separate, but synergistic effects mediated by these two brain regions. The MSDB, placed in the middle of the basal forebrain, receives inputs from other subcortical nuclei involved in oscillatory activity such as the SUM, LH, NI, and Raphe nucleus (Raisman, 1966; Swanson and Cowan, 1979; Ang et al., 2017). They modulate MSDB activity and play a role in the generation or suppression of theta oscillations in the HPC. The LS is also displaying neuronal firing coupled to the theta rhythm (Korotkova et al., 2018). While the MSDB is the major input source to the HPC, LS is one of its most relevant subcortical outputs. Speed-dependent activity has been related to all of these areas with a major difference: MSDB possess pacemaker cells that drive HPC theta even prior to movement initiation (Fuhrmann et al., 2015), while LS theta-locked firing depends on HPC activity (Bender et al., 2015), raising the possibility of a tripartite circuit. In this view, MSDB activation drives HPC oscillations that, in turn, sends the information to the LS about the ongoing motor activity. A recent review on the LS places this structure as a nexus for mood, motivation and movement, postulating its key role in evaluating changes in valence as the result of an animal action (Wirtshafter and Wilson, 2021). This will allow the animal to update its decision whether to act or not depending on the external, context-dependent inputs coming from the HPC and on its internal motivation computed in the LS, based on information flowing from the VTA and other limbic structures. However, also the MSDB has been described as a key element responsible for movement related activity [as MSDB VGluT2^+^ neurons are sufficient to initiate locomotion (Fuhrmann et al., 2015)], motivation [given the increase in self-stimulation of the animals when activating the MSDB (Cazala et al., 1998; Gavello-Baudy et al., 2008)], and mood [see the anxiolytic and anxiogenic effects described after MSDB manipulation (Adhikari et al., 2011; Jiang et al., 2018; Mikulovic et al., 2018)]. It is not surprising that these two regions, given the close anatomical connection and the similar physiological role, also share common effects. The major difference can be found in their connectivity and their cell population. The MSDB intensively projects to the HPC driving theta and sending speed-related information to the whole HPC formation [and possibly sending collaterals to the LS too (Tsanov, 2018)]. It is also highly connected with key structures involved in the animal’s survival (hypothalamic, midbrain, and brainstem regions) and, given its position on the path of the medial forebrain bundle, is closely linked to locomotion (Sinnamon et al., 1984). Moreover, MSDB contains excitatory and modulatory cell types (ChAT and VGluT2) while the LS is mostly comprised of GABAergic interneurons. Undoubtedly, LS has historically been more connected to mood, being involved in the so called septal rage and given the dense projections to the VTA and other areas linked to reward and motivation. Taken together, these observations allow to speculate about a highly interconnected circuit linking MSDB, HPC, and LS involved in movement, speed regulation, and motivation. However, to fully understand this circuit, future studies should explore the directions of the interconnectivity between MSDB and LS, as well as its physiological role.

## Future Directions and Open Questions

We here reviewed the work that has been done so far to disentangle the role of the MSDB cell populations and their projections in relation to locomotion. The rather recent discovery of the VGluT2^+^ population in the MSDB and the yet little work that has been conducted in studying MSDB inputs outside the HPC formation, opens up a large number of questions. What is the role of the GABAergic projections outside the septum? How do VGluT2^+^ neurons drive locomotion? What is the intra-septal connectivity and how do the different population interact with each other? Do more specific cell-types exist among the previously genetically-defined MSDB neurons? What is the relationship between MSDB and LS during behaviour? And what is the physiological role of the MSDB during locomotion in light of the recent findings?

To answer these questions novel tools and techniques have been developed. On one hand, the study of behavioural correlates linked to neural activity is giving previously unimaginable insights. The possibility to look with sub-second resolution at communities and transitions between different behavioral states allows to correlate single cell firing and oscillations to the animals’ action with an unprecedented time resolution (Hong et al., 2015; Wei and Kording, 2018; Luxem et al., 2020; Dunn et al., 2021; Hausmann et al., 2021). Moreover, unsupervised approaches based on machine learning algorithms to score behaviour are replacing manual scoring, which is intrinsically prone to subjective biases and therefore produces results that are hard to compare between studies. On the other hand, development of new genetic tools like faster calcium indicators [jGCaMP8 (Zhang et al., 2020)], more specific opsins to control excitation and inhibition [Opn3 (Mahn et al., 2021), BiPOLES (Vierock et al., 2020)], and novel proteins to detect neuromodulator activity [dLight (Patriarchi et al., 2018), iAChSnFR (Borden et al., 2020), GRAB_NE_ (Feng et al., 2019)], allow to study neuronal dynamics and manipulate cell-type specific neurons with a higher temporal and spatial resolution. Finally, further developments in technologies like freely moving and wireless miniscopes (Aharoni et al., 2019), GRIN lenses and *post hoc* recovery of imaged neurons (Xu et al., 2020), are now giving access to studies that were a technological challenge some decades ago.

Employing all these newly available tools to answer the questions above will shed a novel light on the role of MSDB in a behaviour-specific manner.

## Conclusion

Medial Septum and diagonal Band of Broca has received substantial attention from the field, mainly due to the fact that its lesion or inhibition leads to the abolishment of theta rhythm in the HPC. While early studies emphasised the role of MSDB in locomotion, this aspect of its function has been somewhat neglected in the last years or solely indirectly studied in relation to the theta rhythm. In this review, we have discussed the role of MSDB circuit manipulation, focussing on locomotion as a behavioural readout. We argue that, although the vast manipulation of MSDB circuits leads to an effect on locomotion-related behaviour, the motivation for this type of movements can be very diverse (Figure 1). For example, cholinergic neurons in MSDB are mainly involved in anxiety-related locomotion and action valence, GABAergic neurons seem to regulate aversive behaviours, while glutamatergic neurons are the only ones whose activation leads to an immediate motor response. As these three different cell populations project to different brain regions with very diverse functions (Figure 2), we suggest that future studies should rely on novel technologies as well as computational tools to disentangle specific MSDB cell types role in relation to their projection patterns and their behavioural relevance.

## Author Contributions

Both authors listed have made a substantial, direct and intellectual contribution to the work, and approved it for publication.

## Conflict of Interest

The authors declare that the research was conducted in the absence of any commercial or financial relationships that could be construed as a potential conflict of interest.
